# Mass spectrometric investigation of amorphous Ga-Sb-Se thin films

**DOI:** 10.1038/s41598-019-46767-8

**Published:** 2019-07-15

**Authors:** Ravi Mawale, Tomáš Halenkovič, Marek Bouška, Jan Gutwirth, Virginie Nazabal, Pankaj Lochan Bora, Lukáš Pečinka, Lubomír Prokeš, Josef Havel, Petr Němec

**Affiliations:** 1 Department of Graphic Arts and Photophysics, Faculty of Chemical Technology, University of Pardubice, Studentská 573, 53210 Pardubice, Czech Republic; 2 Institut des Sciences Chimiques de Rennes, UMR-CNRS 6226, Equipe Verres et Céramiques, Université de Rennes 1, 35042 Rennes, France; 3 Department of Chemistry, Faculty of Science, Masaryk University, Kamenice 5, 625 00 Brno, Czech Republic; 4 CEITEC-Central European Institute of Technology Masaryk University, Kamenice 5, 625 00 Brno, Czech Republic; 5 Department of Physical Electronics, Faculty of Science, Masaryk University, Kotlářská 2, 61137 Brno, Czech Republic; 6 CEPLANT, R&D Centre for Low-Cost Plasma and Nanotechnology Surface Modification, Masaryk University, Kotlářská 2, 61137 Brno, Czech Republic

**Keywords:** Surfaces, interfaces and thin films, Mass spectrometry

## Abstract

Amorphous chalcogenide thin films are widely studied due to their enhanced properties and extensive applications. Here, we have studied amorphous Ga-Sb-Se chalcogenide thin films prepared by magnetron co-sputtering, via laser ablation quadrupole ion trap time-of-flight mass spectrometry. Furthermore, the stoichiometry of the generated clusters was determined which gives information about individual species present in the plasma plume originating from the interaction of amorphous chalcogenides with high energy laser pulses. Seven different compositions of thin films (Ga content 7.6–31.7 at. %, Sb content 5.2–31.2 at. %, Se content 61.2–63.3 at. %) were studied and in each case about ~50 different clusters were identified in positive and ~20–30 clusters in negative ion mode. Assuming that polymers can influence the laser desorption (laser ablation) process, we have used parafilm as a material to reduce the destruction of the amorphous network structure and/or promote the laser ablation synthesis of heavier species from those of lower mass. In this case, many new and higher mass clusters were identified. The maximum number of (40) new clusters was detected for the Ga-Sb-Se thin film containing the highest amount of antimony (31.2 at. %). This approach opens new possibilities for laser desorption ionization/laser ablation study of other materials. Finally, for selected binary and ternary clusters, their structure was calculated by using density functional theory optimization procedure.

## Introduction

Chalcogenide glasses (in bulk, fiber, and thin film forms) are fascinating materials due to their excellent properties and wide applications^1^ such as photolithography, holography, for optical switching, laser written waveguides, photonic crystals, optical phase change memories, etc.^2–8^. In recent years amorphous chalcogenides and their thin films are broadly used for optical lenses, demultiplexers, filters, deflectors, Bragg mirrors, couplers, waveguides^9^, for the fabrication of large area photodiode arrays, solar selective coatings, solar cells, photoconductors, sensors, phase change memories, etc.^1,10–14^.

The thin films of amorphous chalcogenides are fabricated using numerous deposition techniques. Currently, the most commonly used deposition methods are pulsed laser deposition^15,16^, radio-frequency magnetron sputtering^17,18,19^, thermal evaporation^20^, chemical vapor deposition^21,22^, etc. Nowadays, these methods are used to prepare high-quality thin films from different binary, ternary, or quaternary chalcogenide glass systems.

Expecting that germanium can be an alternative to arsenic (as a typical, but toxic glass network former), the chalcogenide glasses from the binary Ge-Se system were widely studied to reveal their physical properties (transparency window in IR, (non)linear refractive index, etc.). Moreover, the incorporation of antimony to Ge-Se glasses helps to enhance the glass forming ability and tailor their physical properties^23,24^.

Another approach exploits Ga as a prospective glass network former in Ga-Sb-S^25^, Ga-Sb-Se^26^, Ga-La-S^27,28^, or Ga-Na-S^29^ systems. Among them, considerable attention was recently paid to Ga-Sb-S glasses focusing on their doping with rare earth ions^30,31^. It is well known that the substitution of sulfur by selenium strongly broadens the transmission window of chalcogenide glasses in the infrared range. Therefore, Lecomte *et al*. devoted lately their research to study Ga-Sb-Se glasses transparent up to 18 µm^26^. Moreover, Lu *et al*. studied Ga-Sb-Se chalcogenide system in their bulk as well as thin film forms for the phase change characteristics. According to their results, Ga-Sb-Se materials exhibit outstanding data retention ability, small density change during crystallization, and fast crystallization speed which indicates their great potential for phase change memory applications^32,33^. Therefore, the Ga-Sb-Se chalcogenide glasses and thin films can be considered as a promising alternative for germanium containing ones; however, these materials and their properties are reported in the literature only rarely.

Previous studies evidenced that the laser desorption ionization (LDI) or laser ablation (LA) Time-of-Flight mass spectrometry (TOFMS) is a useful technique for the generation and identification of clusters of binary, ternary or quaternary chalcogenide glasses or their thin films, present in the plasma as a consequence of high energy pulsed laser irradiation^34–36^.

Here, we investigate Ga-Sb-Se amorphous chalcogenide thin films. The thin layers were fabricated via rf magnetron co-sputtering, which allows to obtain thin films with various composition by only adjusting electrical power applied on individual cathodes. This method of thin films fabrication thus enables a cost-effective way to study compositional dependencies of material’s properties. Moreover, co-sputtering allows synthesizing amorphous thin films with compositions inaccessible as glasses in bulk form, i.e. films with compositions outside the glass-forming region of corresponding bulk counterparts. The fabricated films were characterized in detail using LA TOFMS analysis. Further, the clusters generated from parafilm coated and non-coated Ga-Sb-Se thin films were compared. For selected binary and ternary species, their structure was predicted via density functional theory calculations. The resulting information provides a better understanding of the materials fragments present in the plasma, generated via the pulsed laser irradiation of the solid-state material.

## Results and Discussion

The surface characterization of the fabricated thin films was carried out by using SEM. The morphology of the thin layers surface is of good quality as shown by an example of SEM micrograph given in Supplementary Fig. S1. The chemical composition data of thin films, as obtained by EDS when averaging at three measured positions (Table 1), indicate that by using rf power 15–25 and 5–15 W for Ga_2_Se_3_ and Sb_2_Se_3_, respectively, the co-sputtered films cover a broad range of compositions: 7.6–31.7 at. % of Ga and 5.2–31.2 at. % of Sb, while the content of Se remains fairly constant (61.2–63.3 at. %) and in good accordance with nominal composition (60 at. %). XRD patterns revealed that all the deposited films are amorphous. The thickness of the thin films is in the range 560–780 nm (±2 nm).

**Table 1:** Thin films identification, rf sputtering power used on individual cathodes, and chemical composition of the fabricated Ga-Sb-Se thin films determined using EDS analysis (±1 at. %).

Sample name	rf power (W)		Chemical composition (at. %)		
	Ga_2_Se_3_	Sb_2_Se_3_	Ga	Sb	Se
A	25	5	31.7	5.2	63.1
B	20	5	30.3	6.9	62.8
C	20	8	19.2	17.5	63.3
D	20	10	16.7	21.7	61.6
E	20	12	13.6	24.7	61.7
F	20	15	10.9	27.6	61.5
G	15	15	7.6	31.2	61.2

Initially, the Ga-Sb-Se thin films of different compositions (samples labeled as A-G, Tab. 1) fabricated via rf magnetron co-sputtering were characterized by LA TOFMS in both, positive and negative ion modes. The mass spectra were recorded by systematically increasing the laser energy and the threshold energies for the ionization were determined for each sample. The ‘optimal’ mass spectra with sufficient mass resolution and the highest number of clusters were recorded at 160 and 140 a.u. for positive and negative ion modes, respectively, and used for further data processing. For the positive ion mode, the threshold energy where the ionization gets started was found to be 120 a.u., while for the negative ion mode it was 110 a.u. It was observed that for parafilm coated thin films the threshold energy was higher than for the non-coated thin films.

**Figure 1:**
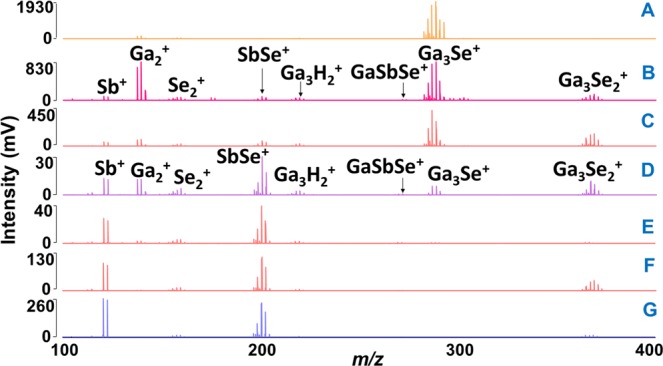
Comparison of mass spectra obtained from Ga-Sb-Se thin films in 100–400 *m/z* range. Conditions: positive ion mode, laser energy 160 a.u. For the sake of clarity, the only description of spectra for two measured samples is given.

**Figure 2:**
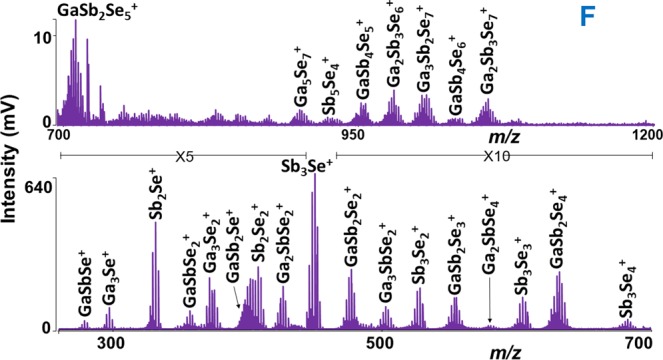
Mass spectrum obtained from non-coated thin film F. Conditions: positive ion mode, laser energy 160 a.u.

**Figure 3:**
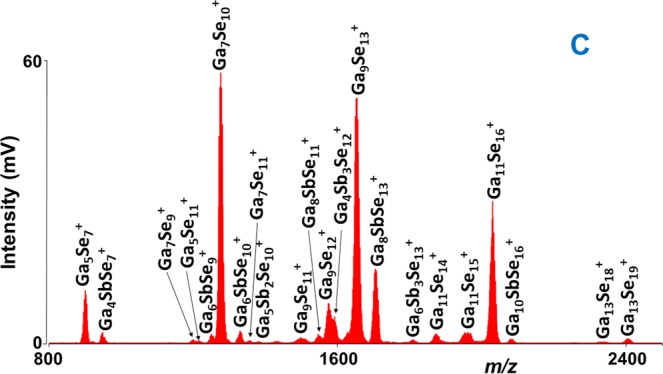
Mass spectrum obtained from parafilm non-coated thin film C. Conditions: positive ion mode, laser energy 160 a.u.

The effect of laser fluence was studied for all the samples (A-G) by recording mass spectra at different laser energies. As mentioned above, after reaching threshold laser energy of 120 a.u. in positive ion mode, the intensity of the mass spectra peaks climbed until 160 a.u. Afterward, the intensity decreased with further increase in laser power. This behavior generally occurs due to the decomposition of the ions at very high laser energies. The stoichiometry of the plasma species was determined by comparison of theoretical and experimental isotopic patterns (Supplementary Fig. S2). For all the thin film samples, the lowest mass ion assigned was Sb^+^ (quadrupole ion trap does not allow the detection of ions below m/z 100, Ga^+^ (69.723) and Se^+^ (78.96) ions maybe formed but they were not detected in the mass spectra). In addition to this one, two other unary Ga_2_^+^ and Se_2_^+^ clusters were detected. Many binary Ga_x_Se_z_^+^ and Sb_y_Se_z_^+^ clusters were also identified. The comparison of mass spectra for all the samples in range m/z 100–400 is given in Fig. 1. In this m/z range of mass spectra mostly unary and binary together with few ternary clusters were detected. Qualitatively, the clusters obtained in this mass range are the same for all the samples; however, their intensities are varied according to the chemical composition of the sample. For all the samples, the lowest mass binary clusters identified (correspond to general formula Ga_x_Se_z_^+^ and Sb_y_Se_z_^+^) were Ga_2_Se^+^ and SbSe^+^, while the highest mass clusters Ga_19_Se_28_^+^ and Sb_5_Se_4_^+^ were detected for samples A and F, respectively. It is worthy to note that no Ga_x_Sb_y_^+^ species were detected. On the other hand, many ternary Ga_x_Sb_y_Se_z_^+^ clusters were observed. As examples, the F and C samples mass spectra in range m/z 250–1200 and 800–2500 are given in Figs 2 and 3, respectively. It was observed that the intensity of the peaks corresponding to the high mass clusters is lower than those detected in the low mass range m/z 100–400. The list of clusters detected by LA TOFMS in positive ion mode for all the samples is given in Supplementary Table S1. For simplicity, the number of binary Ga_x_Se_z_^+^, Sb_y_Se_z_^+^, and ternary Ga_x_Sb_y_Se_z_^+^ clusters detected during LA of each thin film (parafilm coated and non-coated) sample is plotted and shown in Supplementary Fig. S3(a–c). For non-coated thin films, the highest number of Ga_x_Se_z_^+^, Sb_y_Se_z_^+^, and ternary Ga_x_Sb_y_Se_z_^+^ clusters was identified for sample B, E, and F, respectively. For samples A-D, the highest intensity clusters are gallium selenide (Ga_3_Se^+^ or Ga_3_Se_2_^+^), while for the sample E, F, and G, the antimony selenide clusters (Sb_3_Se_2_^+^ or Sb_3_Se^+^) are of highest intensity. Several low-intensity binary Ga_x_Se_z_^+^, Sb_y_Se_z_^+^, and ternary Ga_x_Sb_y_Se_z_^+^ clusters were also detected, but due to the insufficient mass resolution, the stoichiometry of these species was not determined.

**Figure 4:**
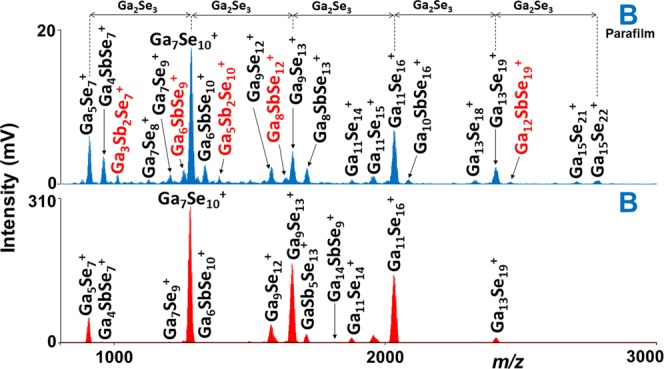
Comparison of mass spectra obtained for sample B with and without the parafilm coating. Conditions: positive ion mode, laser energy 160 a.u.

Secondly, the parafilm coated thin films were examined via LA at similar experimental conditions and the results were compared with the non-coated ones. Many new, especially high mass clusters were identified in case of parafilm coated films. The comparison of mass spectra obtained from parafilm coated and non-coated thin film (sample B) is given in Fig. 4. Generally, one can observe that in most of the cases the intensity of the peaks obtained for non-coated thin films is higher than for coated ones under identical experimental conditions. Similarly to the non-coated thin films, Sb^+^ is the lowest mass species identified for all the samples, while the highest mass cluster was Ga_19_Se_28_^+^ which is detected in samples B, D, E, and F. The highest number of Ga_x_Se_z_^+^ (23) and Sb_y_Se_z_^+^ (9) clusters detected for samples B and C, E, G, respectively (Supplementary Fig S3a,b). Many new ternary Ga_x_Sb_y_Se_z_^+^ clusters were also generated from parafilm coated thin films especially for the samples C-G, which have larger content of antimony (≥17.5 at. %). The highest number of ternary clusters (38) was identified for the parafilm coated thin film G (Supplementary Fig. S3c). An important part of the mass spectrum (m/z 800–5000) obtained for parafilm coated thin film G is presented in Supplementary Fig. S4. A series of peaks such as Ga_5_Se_7_, Ga_7_Se_10_, Ga_9_Se_13_, Ga_11_Se_16,_ etc. are prominently detected and the difference between these main peaks is Ga_2_Se_3_ (c.f. Fig. 4) with the largest cluster of the series as Ga_19_Se_28_^+^.

Considering the MS results in all m/z ranges, the highest intensity peaks for samples A and B (<17.5 at. % of Sb) are Ga_3_Se_2_^+^ and Ga_3_Se^+^, while for the samples C-G (≥17.5 at. % of Sb), the most intensive are Sb_3_Se_2_^+^ and Sb_3_Se^+^ peaks. An overview of all the species detected for coated as well as non-coated Ga-Sb-Se thin films is summarized in Supplementary Table S1. Few other clusters were also observed in MS data, but the stoichiometry of these species was not determined due to the insufficient mass resolution.

Furthermore, for both coated and non-coated Ga-Sb-Se thin films, it was found that the intensities of most of the peaks were varied according to the chemical composition of the thin films. For example, thin films with a higher amount of Ga (samples A, B) have high intensity of Ga_*x*_Se_*z*_^+^ clusters, while the intensities decreased as the amount of Sb increased (samples C-G) and vice-versa for Sb_*y*_Se_*z*_^+^ clusters. To exemplify this, we have selected some clusters which are identified in all the samples and plotted their intensities versus chemical composition (Supplementary Figs S5 and S6). The intensities of the Ga_2_^+^ and Ga_3_Se^+^ species is the highest for samples A and B and then decreases for samples C-G as the amount of Sb particularly increased. In contrary, the intensities of Sb^+^ and SbSe^+^ increased; they are highest for sample G where the content of Ga is lowest (7.6 at. %) and Sb is largest (31.2 at. %). It should be noticed that no significant intensity difference was observed in the case of Se_2_^+^ and ternary GaSbSe^+^ clusters.

**Figure 5:**
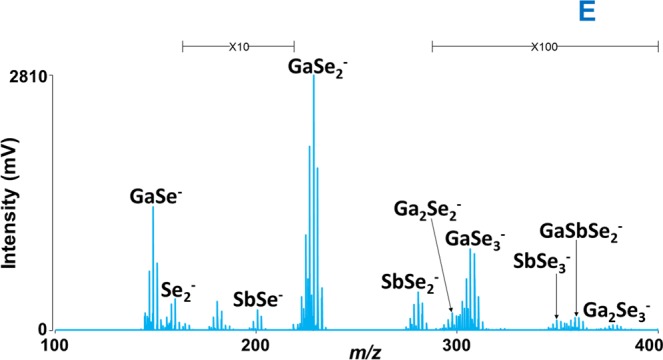
Mass spectrum obtained for parafilm non-coated thin film (sample E). Conditions: negative ion mode, laser energy 140 a.u.

All the thin film samples (coated and non-coated) were also examined via LA TOFMS in negative ion mode; however, the spectra are not that ‘rich’ like in positive ion mode with respect to the number of species detected. The effect of laser energy was studied by recording mass spectra at different laser power. It was observed that the intensity of the peaks grew with the increased laser energy until 140 a.u.; with a further rise of laser energy, the intensity dropped. The ‘optimal’ spectra with sufficient mass resolution and the highest number of peaks were recorded at a laser energy of 140 a.u. The lowest mass species identified (at these experimental conditions) is Se_2_^−^ in all the samples and the highest mass clusters detected was Ga_17_Se_26_^−^ for parafilm coated thin film B. Considering the mass spectra in all *m/z* ranges, the highest intensity peak observed for all the thin films is GaSe_2_^−^_._ An example of a negative ion mode mass spectra in the range of *m/z* 100–400 for sample E is given in Fig. 5. The complete list of negatively charged clusters identified during LA of parafilm coated and uncoated Ga-Sb-Se thin films is given in Supplementary Table S2. Most of the negatively charged clusters detected are found to be different than positively charged ones. Many new and higher mass negatively charged clusters were detected for parafilm coated thin films as compared to non-coated ones. A comparison of negative ion mode mass spectra obtained from parafilm coated and non-coated thin film F is given in Supplementary Fig. S7.

### Effect of parafilm coatings of thin films on mass spectra

LA is basically a destructive method which may generally break the original structure of the studied materials. In the case of amorphous Ga-Sb-Se films, we identified many species generated during LA. As already reported in^35,37^, some of the species might originate from real glass network structure, which makes generally possible to obtain structural information for solid state materials like glasses and their thin films. In our previous study^38^, we have shown that by dispersing the powdered chalcogenide material into a parafilm solution or in polymers (polyvinylpyrrolidone, polyethylene glycol, etc.), resulting mass spectra contained a significantly higher number of identified species present in the plasma plume. Moreover, the parafilm came out to be the most efficient material (among investigated ones) in terms of highest number of clusters detected in mass spectra of binary arsenic chalcogenides (As_2_Ch_3_, Ch = S, Se, Te).

Therefore, in this work, we have used a parafilm to coat Ga-Sb-Se thin films. As a result, many new clusters were identified in both, positive and negative ion modes using the technique depicted above. In positive ion mode, for some thin films, more than 30 new species were detected. The largest number of new clusters (40) was identified for a film with the highest content of antimony (sample G). In negative ion mode, many new clusters were also detected with a maximum of 16 new clusters, again for film G.

One can speculate that among detected new clusters, mostly higher mass clusters can be considered as larger fragments of the original glass network structure. Therefore, one can suggest that to some extent parafilm might reduce the fragmentation of larger parts of the structures into lower fragments. As a result, some more complex structures in the form of higher mass clusters are generated when using parafilm. But, in that case, the number of low mass clusters would be decreased; nevertheless this has not been observed. Therefore, another interpretation might be that the parafilm can play some role within the reactions of highly energetic low mass species present in the plasma plume. These lower mass constituents then might undergo laser ablation synthesis (LAS) and form heavier clusters.

**Figure 6:**
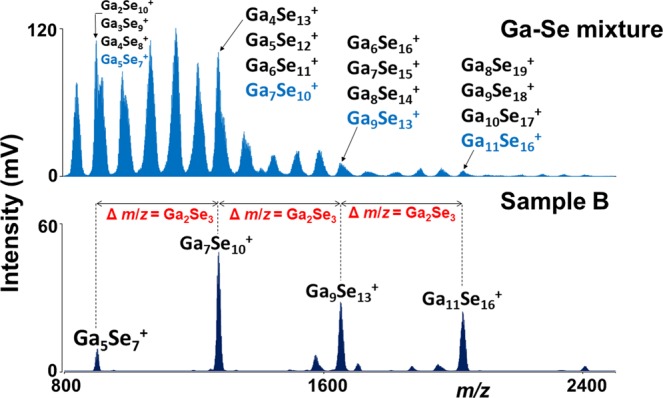
Comparison of clusters generated from a thin film (Sample B) with those from Ga-Se mixture. Conditions: positive ion mode, laser energy 160 and 170 a.u., respectively.

**Figure 7:**
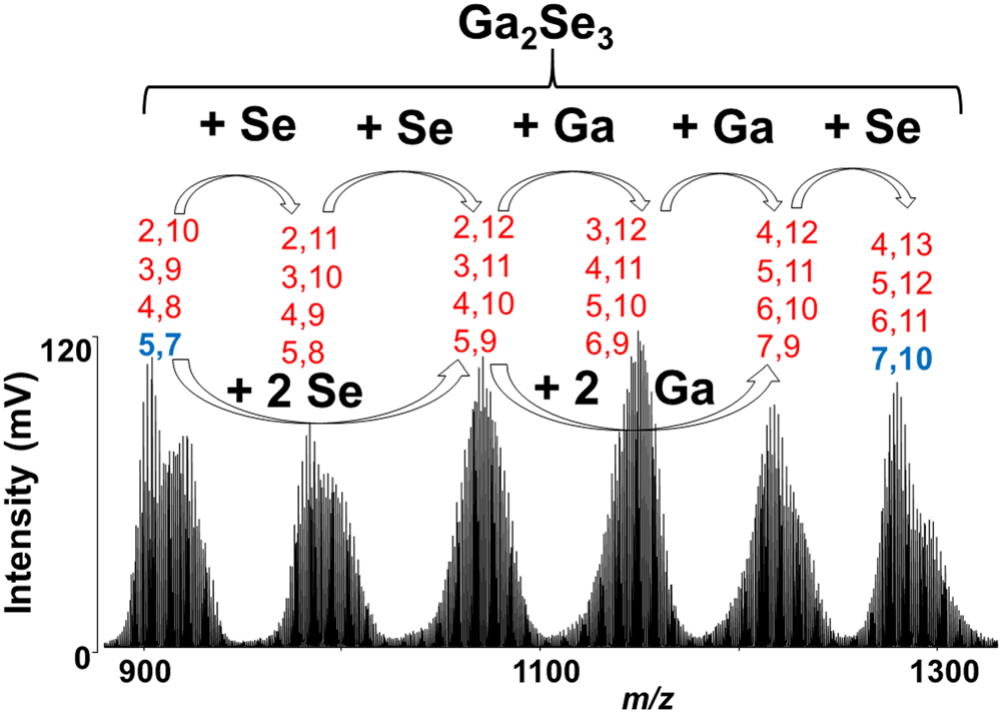
Possible mechanism of high mass Ga_*m*_Se_*n*_ clusters generation. Conditions: positive ion mode, laser energy 170 a.u. The stoichiometry of clusters is given as: Ga,Se.

Both theories are equivalent for the results. Higher mass clusters are formed (i) because of lower fragmentation under the presence of parafilm or (ii) parafilm is inducing synthesis of higher mass clusters from lower ones, it is difficult to distinguish here. We have therefore studied the synthesis of binary Ga_*x*_Se_*z*_ clusters also from the mixture of elements. The mass spectra obtained from Ga:Se mixture and Ga-Sb-Se thin films were compared (Fig. 6). Evidently, main clusters Ga_5_Se_7_^+^, Ga_7_Se_10_^+^, Ga_9_Se_13_^+^, Ga_11_Se_16_^+^, etc. are also detected during LAS from the mixture of elements but there are also many other clusters. One can speculate that the possible mechanism of the formation of the clusters might be step-by-step synthesis from low mass clusters reacting with selenium and/or gallium (Fig. 7).

Indeed, the presence of parafilm probably leads to complex processes including several features resulting in the observation of many species identified in mass spectra. In the absence of the parafilm, some clusters may dissipate fast and are not observed, while under the presence of parafilm, they are stabilized and a higher number of clusters maybe detected. It cannot be excluded that under the presence of parafilm higher mass clusters are generated via LAS reactions from lower mass fragments.

However, there is also another possibility. As demonstrated in^39–41^, laser desorption ionization from the surface of parafilm leads to increased sensitivity. Concluding, all above depicted explanations might be possible, they can proceed simultaneously in lower or higher extent and it is difficult to decide which one prevails.

### Structure of Ga_x_Se_z_, Sb_y_Se_z_, and Ga_x_Sb_y_Se_z_ clusters

TOFMS does not provide direct structural information and thus it is difficult to elucidate the structure of the generated clusters from the mass spectra itself. On the other hand, the structures of many gallium^42–45^, antimony^46–48^, and selenium^49,50^ clusters are known. Apart from the aforementioned structures, only a few structures of the binary gallium selenide and antimony selenide clusters have been reported^51,52^. However, there are extensive laser ablation mass spectrometric results showing that in binary systems Ga-Se and Sb-Se over one hundred Ga_x_Se_z_ and Sb_y_Se_z_ clusters were detected^53,54^.

The structure of most of the clusters generated in this work was not previously reported. Moreover, the number of possible structural isomers highly increases with the growing number of atoms in the clusters.

**Figure 8:**
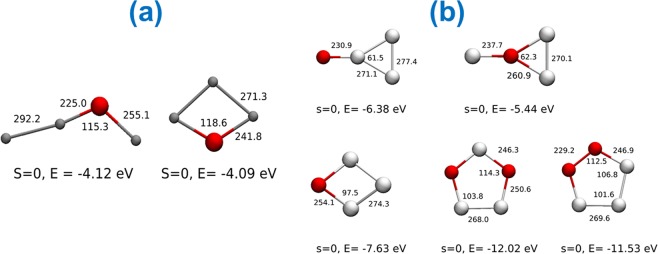
Calculated structural isomers of Ga_3_Se^+^ (**a**), Sb_3_Se^+^ and Sb_3_Se_2_^+^ (**b**). Spin, bond energy in eV, bond lengths in pm, and angles in degree are given. Gray, silver and red balls represent gallium, antimony, and selenium atoms, respectively.

**Figure 9:**
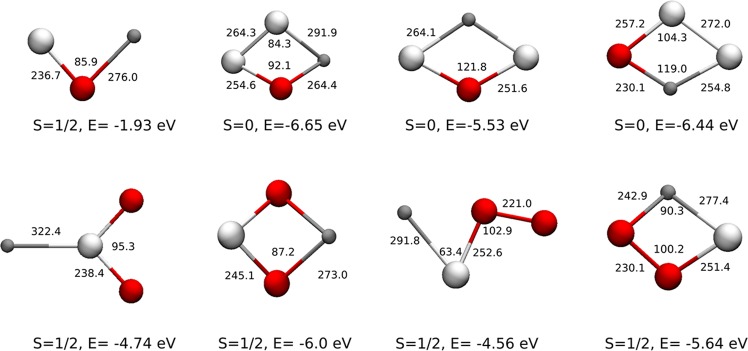
Example of calculated structural isomers of monocationic GaSb_*y*_Se_*z*_ with y and *z* = 1–2. Spin, bond energy in eV, bond lengths in pm, and angles in degree are given. Gray, silver and red balls represent gallium, antimony, and selenium atoms, respectively.

The structural patterns for selected binary and ternary clusters generated during LA of Ga-Sb-Se amorphous thin films were computed by density functional theory (DFT) optimization. A few possible structural isomers which are in the ground state with local minima in the potential energy surface are shown in Figs 8 and 9. The structures with the lowest energy are shown here if more than one stable structures of the same stoichiometry were found. From the calculated data it was observed that for small Ga_*x*_Se_*z*_, Sb_*y*_Se_*z*_, and Ga_*x*_Sb_*y*_Se_*z*_ (*x, y, z* = 1–3) clusters, the molecular geometry does not change much with the charge. The bond lengths and angles of the structure for monocationic, neutral and monoanionic entities differ by only a few percents. As we obtained extensive data in positive ion mode during LA TOFMS, we further provided the calculated molecular data for singly charged positive clusters.

The DFT optimized structures of selected binary and ternary clusters are given in Figs 8 and 9. Optimizations were done first at the lower level of theory using LDA approximation with TZ2P basis set^55^, frozen core, and scalar relativistic ZORA^56–58^ methods followed by a higher level of theory with OPBE^59^ functional and all electron QZ4P basis sets^55^ with scalar relativistic ZORA approximation in ADF package^60,61^ (Version 2017). The unrestricted approach was used in the case of open-shell structures. The frequencies are calculated by using Gaussian software package^62^. The frequencies are computed from the square roots of the force constants. The optimized structures correspond to the “ground state” with local minimum in the potential energy surfaces with no imaginary frequency. The structures with the lowest energy are shown here if more than one stable structures of the same stoichiometry were found. The typical distance from adjacent gallium and selenium nuclei lies between 230–280 pm, while the distance between gallium-gallium nuclei is 270–290 pm. The distance between antimony and selenium nuclei is found between 230–260 pm and for Sb-Sb nuclei it is 260–280 pm. Detailed calculations are needed to generalize the structural features of all the generated clusters; however, this is beyond the scope of this study.

## Conclusions

LA of Ga-Sb-Se amorphous thin films prepared via co-sputtering method from Ga_2_Se_3_ and Sb_2_Se_3_ polycrystalline targets generates many unary, binary and ternary clusters (Ga_*x*_^+/−^, Sb_*y*_^+/−^, Se_*z*_^+/−^, Ga_*x*_Se_*z*_^+/−^, Sb_*y*_Se_*z*_^+/−^, and Ga_*x*_Sb_*y*_Se_*z*_^+/−^). As deposited Ga-Sb-Se thin films with seven different chemical compositions were examined and in each case, approximately 50 clusters were identified in positive ion mode. When parafilm coated Ga-Sb-Se thin films were studied, many new and high mass clusters were identified. The highest number (40) of new clusters was detected in positive ion mode for the thin film containing the largest amount of antimony. This indicates that parafilm is playing an important role during the LA process either reducing the destruction of the original amorphous network structure during the interaction of laser pulses with amorphous material or promoting the laser ablation synthesis of heavier species from low mass ones. Also, it is possible that detection from the parafilm surface is more sensitive. To get insight into the structure of the observed species, some DFT calculations were performed.

Concluding, LA TOFMS is found to be an important and useful analytical technique to study the amorphous chalcogenide thin films in terms of identification of species present in the plasma phase when the materials are exposed to the laser pulses. The stoichiometry of the species might help for partial structural characterization of thin films. Lastly, the parafilm as a material increasing the number of identified species in the plasma can be used to widen the applicability of the LA for other materials and thin films.

## Experimental

### Chemicals and materials

Gallium, selenium, methanol, acetone, and acetonitrile were purchased from Sigma-Aldrich (Steinheim, Germany). Polycrystalline 2″ 99.999% Ga_2_Se_3_ and Sb_2_Se_3_ sputtering targets were purchased from ALB Materials, Inc., Henderson, NV, USA. Xylene (a mixture of isomers) was purchased from Mikrochem Spol. s.r.o. (Pezinok, Slovak Republic) and parafilm from Pechiney Plastic Packaging (Chicago, IL, USA). Micro-90 (cleaning agent) was from Kratos (Manchester, UK). Silicon wafers used as substrates for the thin films depositions were purchased from ON SEMICONDUCTORS (Czech Republic). Deionized water was distilled once in glass and then double distilled from a quartz apparatus Heraeus Quarzschmelze (Hanau, Germany) to produce ultrapure water. All the other reagents were of analytical grade purity.

### Methods

#### Ga-Sb-Se thin films

Amorphous thin films from Ga-Sb-Se system were fabricated using conventional rf (13.56 MHz) magnetron co-sputtering technique. The chemical composition of the thin films was varied by changing the rf power (up to 25 W) on the two cathodes (Table 1). The experimental conditions of the co-sputtering deposition process are described elsewhere^17^.

#### Ga:Se mixture

Gallium-selenium mixture was prepared as suspension (~1 mg/mL) in acetonitrile by mixing liquid gallium with selenium powder in a molar ratio of 1:1 and applying ultrasonication (15 min). This suspension was used for MS analysis.

### Scanning electron microscopy with energy dispersive X-ray spectroscopy and X-ray diffraction

A scanning electron microscope (SEM) with an energy dispersive X-ray analyzer (EDS, TESCAN VEGA 3 EasyProbe) was used for the study of surface morphology and determination of chemical composition of fabricated materials. The uncertainty of EDS measurements for the studied films is ±1 at. %. Typically, the EDS measurements were performed at 3 spots per sample and averaged. X-ray diffraction (XRD) technique (D8-Advance diffractometer, Bruker AXS, Germany) was exploited to determine the structure of co-sputtered thin films using Bragg–Brentano θ–θ geometry with CuKα radiation and a secondary graphite monochromator. The diffraction angles were measured at room temperature from 5 to 65° (2θ) within 0.02° steps.

### LA TOF mass spectrometry

The cleaning of the target plate was performed according to the Shimadzu target cleaning protocol. It includes initial cleaning with water, followed by sonication in Micro-90 cleaning agent solution for 15–20 min and then rinsing several times with water, acetone, and methanol. Finally, the target was air dried and kept overnight under vacuum in MS instrument and then used for measurements.

Thin films were fixed on the target plate using an adhesive tape. A part of the thin film was coated manually with a parafilm solution prepared by dissolving a piece of parafilm (1 cm × 1 cm) in xylene (1 ml). The thickness of the parafilm layer on studied films was approximately 100 μm. The coated and non-coated parts of the thin film were then examined using LA TOF mass spectrometry. The absorption coefficient of used parafilm at the wavelength of laser exploited for LA TOFMS (337 nm) is ~95 ± 20 cm^−1^ as calculated from its transmission spectrum.

AXIMA Resonance mass spectrometer from Kratos Analytical Ltd. (Manchester, UK) was used to record the mass spectra. The instrument details are given elsewhere^38^. Mass spectra were acquired in both, positive and negative ion modes by accumulating the data from at least 2000 laser shots. The peaks with sufficient mass resolution and intensity values higher than 1 mV (3 sigma of noise level) are considered relevant. For both modes, each *m/z* range was calibrated externaly using red phosphorus clusters^63^, while the accuracy achieved was below ±20 mDa.

Theoretical isotopic patterns were calculated using Launchpad software (Kompact version 2.9.3, 2011) from Kratos Analytical Ltd. (Manchester, UK). The examples of possible cluster structures were computed via DFT calculations using scalar relativistic ZORA approximation in ADF package (Version 2017)^60,61^.

## Supplementary information

Mass spectrometric investigation of amorphous Ga-Sb-Se thin films

## Data Availability

The datasets generated and analysed during the current study are available from the corresponding author on reasonable request.

## References

[1] Adam, J.-L. & Zhang, X. *Chalcogenide glasses: Preparation, properties and applications*. (Woodhead Publishing limited, 2014).

[2] Song S (2009). Spin-coating of Ge_23_Sb_7_S_70_ chalcogenide glass thin films. J. Non. Cryst. Solids.

[3] Song X, Zhou W, Liu X, Gu Y, Zhang S (2017). Layer-controlled band alignment, work function and optical properties of few-layer GeSe. Phys. B Condens. Matter.

[4] Wang Y (2017). Composition dependences of refractive index and thermo-optic coefficient in Ge-As-Se chalcogenide glasses. J. Non. Cryst. Solids.

[5] Qiao C (2018). Evolution of short- and medium-range order in the melt-quenching amorphization of Ge_2_Sb_2_Te_5_. J. Mater. Chem. C.

[6] Xu M, Cheng YQ, Sheng HW, Ma E (2009). Nature of atomic bonding and atomic structure in the phase-change Ge_2_Sb_2_Te_5_ glass. Phys. Rev. Lett..

[7] Xu K, Miao X, Xu M (2019). The structure of phase-change chalcogenides and their high- pressure behavior. Phys. Status Solidi RRL.

[8] Guo YR (2018). Structural signature and transition dynamics of Sb_2_Te_3_ melt upon fast cooling. Phys. Chem. Chem. Phys..

[9] Molnar S, Bohdan R, Takats V, Kaganovskii Y, Kokenyesi S (2018). Viscosity of As_20_Se_80_ amorphous chalcogenide films. Mater. Lett..

[10] Mehta N (2006). Applications of chalcogenide glasses in electronics and optoelectronics: A review. J. Sci. Ind. Res..

[11] Raoux S, Wełnic W, Lelmini D (2010). Phase change materials and their application to nonvolatile memories. Chem. Rev..

[12] Eggleton BJ, Luther-Davies B, Richardson K (2011). Chalcogenide photonics. Nat. Photonics.

[13] Zakery A, Elliott SR (2003). Optical properties and applications of chalcogenide glasses: a review. J. Non. Cryst. Solids.

[14] Zakery, A. & Elliott, S. *Optical Nonlinearities in Chalcogenide Glasses and their Applications*. **135**, (Springer Berlin Heidelberg, 2007).

[15] Olivier M (2015). Photosensitivity of pulsed laser deposited Ge-Sb-Se thin films. Opt. Mater. Express.

[16] Bouška M (2016). Pulsed laser deposited GeTe-rich GeTe-Sb_2_Te_3_*thin films*. Sci. Rep..

[17] Halenkovič T (2018). Amorphous Ge-Sb-Se thin films fabricated by co-sputtering: Properties and photosensitivity. J. Am. Ceram. Soc..

[18] Baudet E (2017). Experimental design approach for deposition optimization of RF sputtered chalcogenide thin films devoted to environmental optical sensors. Sci. Rep..

[19] Verger F (2013). RF sputtered amorphous chalcogenide thin films for surface enhanced infrared absorption spectroscopy. Opt. Mater. Express.

[20] Mandal D (2018). Intensity mediated change in the sign of ultrafast third-order nonlinear optical response in As_2_S_2_ thin films. Opt. Lett..

[21] Benjamin SL (2015). Chemical vapour deposition of antimony chalcogenides with positional and orientational control: Precursor design and substrate selectivity. J. Mater. Chem. C.

[22] Abrutis A (2008). Chemical vapor deposition of chalcogenide materials for phase-change memories. Microelectron. Eng..

[23] Němec P (2014). Optical properties of (GeSe_2_)_100-*x*_(Sb_2_Se_3_)_*x*_ glasses in near- and middle-infrared spectral regions. Mater. Res. Bull..

[24] Wei WH, Wang RP, Shen X, Fang L, Luther-Davies B (2013). Correlation between structural and physical properties in Ge-Sb-Se glasses. J. Phys. Chem. C.

[25] Yang A (2016). Ga-Sb-S chalcogenide glasses for mid-infrared applications. J. Am. Ceram. Soc..

[26] Lecomte A, Nazabal V, Le Coq D, Calvez L (2018). Ge-free chalcogenide glasses based on Ga-Sb-Se and their stabilization by iodine incorporation. J. Non. Cryst. Solids.

[27] Mairaj, A. K. *et al*. Advances in gallium lanthanum sulphide glass for optical fiber and devices. In *SPIE 4204, Fiber Optic Sensor**Technology II* (eds Culshaw, B., Harrington, J. A., Marcus, M. A. & Saad, M.) 278–286, 10.1117/12.417421 (2001).

[28] Schweizer T, Hewak DW, Samson BN, Payne DN (1996). Spectroscopic data of the 1.8-, 2.9-, and 4.3-mm transitions in dysprosium-doped gallium lanthanum sulfide glass. Opt. Lett..

[29] Tawarayama H (2000). Optical amplification at 1.3 μm in a praseodymium-doped sulfide-glass fiber. J. Am. Ceram. Soc..

[30] Li G (2016). Er^3+^ doped and Er^3+^/Pr^3+^ co-doped gallium-antimony-sulphur chalcogenide glasses for infrared applications. Opt. Mater. Express.

[31] Jiao Q (2017). Effect of gallium environment on infrared emission in Er^3+^-doped gallium-antimony-sulfur glasses. Sci. Rep..

[32] Lu Y (2014). Phase change characteristics of Sb-rich Ga-Sb-Se materials. J. Alloys Compd..

[33] Lu Y (2011). Ga-Sb-Se material for low-power phase change memory. Appl. Phys. Lett..

[34] Pangavhane SD (2014). Laser desorption ionization time-of-flight mass spectrometry of erbium-doped Ga-Ge-Sb-S glasses. Rapid Commun. Mass Spectrom..

[35] Pangavhane S, Němec P, Wagner T, Janca J, Havel J (2010). Laser desorption ionization time-of-flight mass spectrometric study of binary As-Se glasses. Rapid Commun. Mass Spectrom..

[36] Šútorová K (2016). Laser desorption ionization time-of-flight mass spectrometry of glasses and amorphous films from Ge-As-Se system. J. Am. Ceram. Soc..

[37] Houška J (2009). Laser ablation of AgSbS_2_ and cluster analysis by time-of-flight mass spectrometry. Rapid Commun. Mass Spectrom..

[38] Mawale RM (2017). Laser desorption ionization of As_2_Ch_3_ (Ch = S, Se, and Te) chalcogenides using quadrupole ion trap time-of-flight mass spectrometry: A comparative study. J. Am. Soc. Mass Spectrom..

[39] Urban PL, Amantonico A, Zenobi R (2011). Lab-on-a-plate: Extending the functionality of MALDI-MS and LDI-MS targets. Mass Spectrom. Rev..

[40] Hung KC, Rashidzadeh H, Wang Y, Guo B (1998). Use of paraffin wax film in MALDI-ToF analysis of DNA. Anal. Chem..

[41] Wang J, Chen R, Ma M, Li L (2008). MALDI MS sample preparation by using paraffin wax film: Systematic study and application for peptide analysis. Anal. Chem..

[42] Zhao Y, Xu W, Li Q, Xie Y, Schaefer HF (2004). Gallium clusters Ga_*n*_ (n = 1-6): Structures, thermochemistry, and electron affinities. J. Phys. Chem. A.

[43] Núñez S, López JM, Aguado A (2012). Neutral and charged gallium clusters: Structures, physical properties and implications for the melting features. Nanoscale.

[44] Drebov N, Weigend F, Ahlrichs R (2011). Structures and properties of neutral gallium clusters: A theoretical investigation. J. Chem. Phys..

[45] Gong XG, Tosatti E (1992). Structure of small gallium clusters. Phys. Lett. A.

[46] Geusic ME, Freeman RR, Duncan MA (1988). Neutral and ionic clusters of antimony and bismuth: A comparison of magic numbers. J. Chem. Phys..

[47] Sundararajan V, Kumar V (1995). *Ab initio* molecular dynamics study of antimony clusters. J. Chem. Phys..

[48] Jones RO, Ahlstedt O, Akola J, Ropo M (2017). Density functional study of structure and dynamics in liquid antimony and Sb_*n*_ clusters. J. Chem. Phys..

[49] Alparone A (2012). Density functional theory Raman spectra of cyclic selenium clusters Se_*n*_ (n = 5–12). Comput. Theor. Chem..

[50] Hohl D, Jones RO, Car R, Parrinello M (1987). The structure of selenium clusters Se_3_ to Se_8_. Chem. Phys. Lett..

[51] Gurin, V., Shpotyuk, O. & Boyko, V. Calculation of small arsenic and antimony chalcogenide clusters with an application to vitreous chalcogenide structure. 6 arXiv.1504.00246v1 (2015).

[52] Ghalouci L (2013). First principle investigation into hexagonal and cubic structures of Gallium Selenide. Comput. Mater. Sci..

[53] Pečinka L, Lubomir P, Havel J (2019). Gallium selenide clusters generated via laser desorption ionisation time-of-flight quadrupole ion trap mass spectrometry. Rapid Commun. Mass Spectrom..

[54] Fei H, Prokes L, Havel J (2019). Laser ablation generation of antimony selenide clusters: laser desorption ionization (LDI) quadrupole ion trap time of flight mass spectrometry. J. Am. Soc. Mass Spectrom..

[55] van Lenthe E, Baerends EJ (2003). Optimized Slater-type basis sets for the elements 1-118. J. Comput. Chem..

[56] van Lenthe E, Baerends EJ, Snijders JG (1993). Relativistic regular two-component Hamiltonians. J. Chem. Phys..

[57] van Lenthe E, Baerends EJ, Snijders JG (1994). Relativistic total energy using regular approximations. J. Chem. Phys..

[58] van Lenthe E, Ehlers A, Baerends E (1999). Geometry optimizations in the zero order regular approximation for relativistic effects. J. Chem. Phys..

[59] Swart M, Ehlers AW, Lammertsma K (2004). Performance of the OPBE exchange-correlation functional. Mol. Phys..

[60] Velde GTE (2001). Chemistry with ADF. J. Comput. Chem..

[61] Guerra CF, Snijders JG, Velde G, Baerends EJ (1998). Regular article Towards an order- N DFT method. Theor. Chem. Acc..

[62] Frisch, M. J. *et al*. *Gaussian 16, Revision B.01*. Gaussian, Inc., (Wallingford CT, 2016).

[63] Sladkova K, Houska J, Havel J (2009). Laser desorption ionization of red phosphorus clusters and their use for mass calibration in time-of-flight mass spectrometry. Rapid Commun. Mass Spectrom..

