# Supplementary material for: Mass spectrometric investigation of amorphous Ga-Sb-Se thin films
Source: Sci Rep. 2019 Jul 15;9:10213. doi: 10.1038/s41598-019-46767-8 (PMC6629872; doi:10.1038/s41598-019-46767-8)
Supplement: Supplementary file 1 — Mass spectrometric investigation of amorphous Ga-Sb-Se thin films [file 41598_2019_46767_MOESM1_ESM.pdf]

Supplementary information:

Mass spectrometric investigation of amorphous Ga-Sb-Se thin films

Ravi Mawale, Tomáš Halenkovič, Marek Bouška, Jan Gutwirth, Virginie Nazabal, Pankaj Lochan Bora, Lukáš Pečinka, Lubomír Prokeš, Josef Havel, and Petr Němec*

*Corresponding author: petr.nemec@upce.cz (Petr Němec)

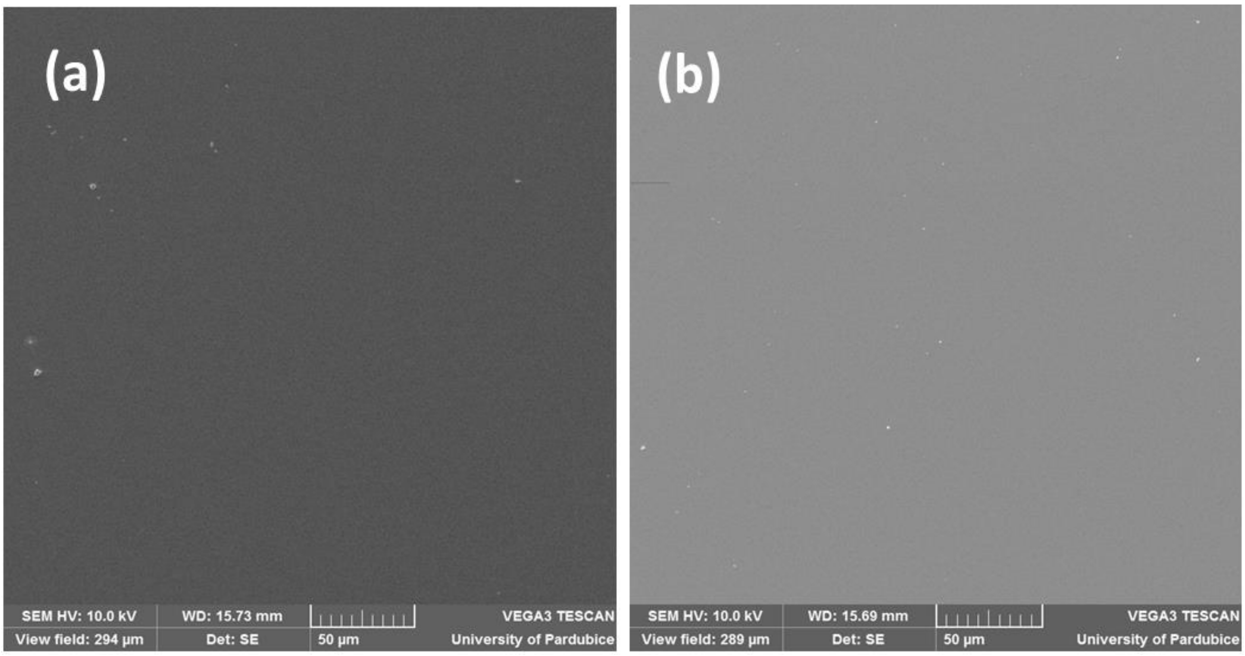

Supplementary Figure S1: SEM images of Ga-Sb-Se thin films a) sample F and b) sample G.

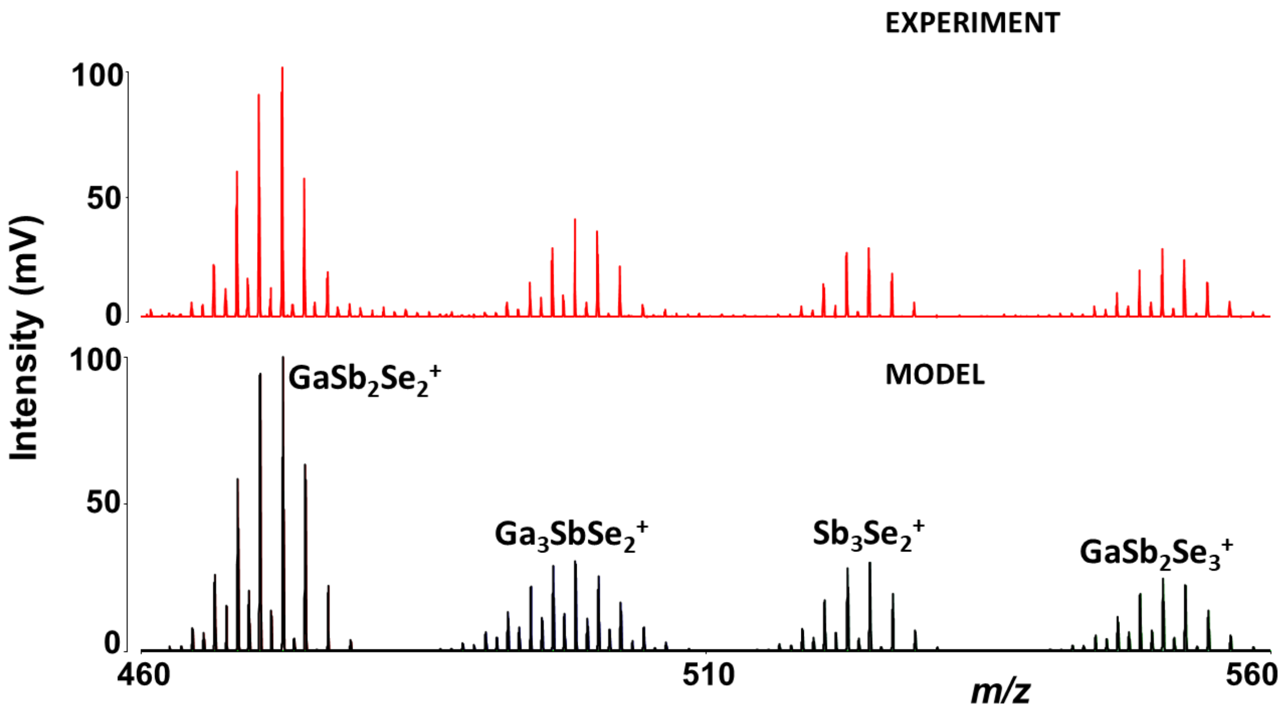

Supplementary Figure S2: Comparison of experimental and theoretical model mass spectra obtained from parafilm coated thin films of sample E concerning the formation of $GaSb_2Se_2^+$, $Ga_3SbSe_2^+$, $Sb_3Se_2^+$, and $GaSb_2Se_3^+$ clusters. Conditions: positive ion mode, laser energy 180 a.u.

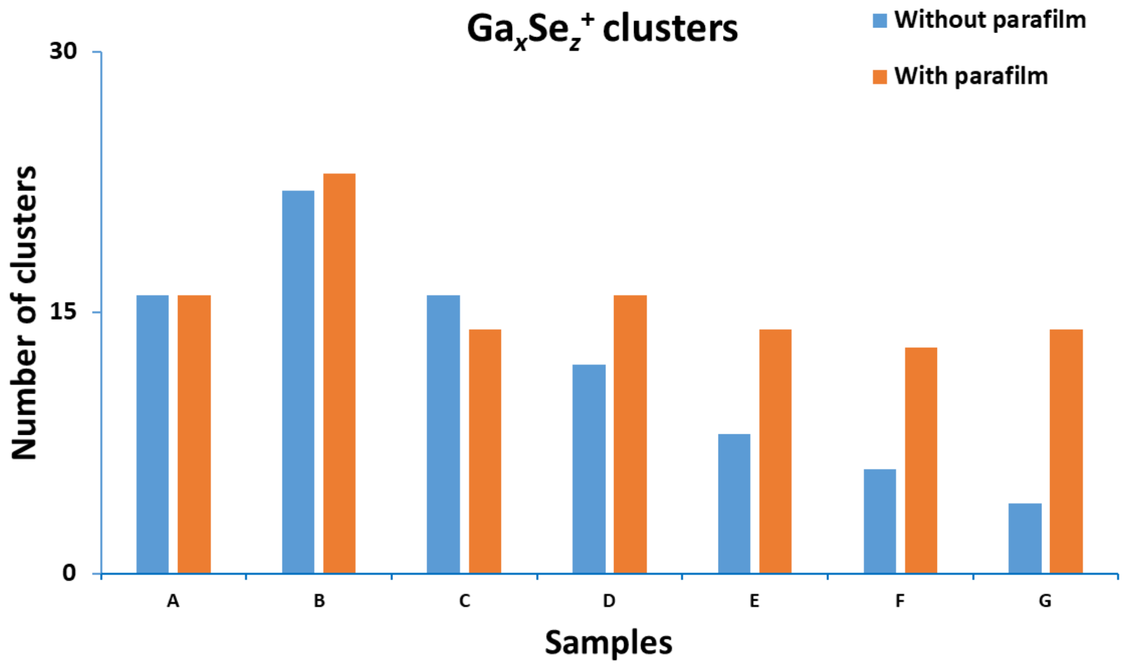

Supplementary Figure S3a: The number of binary $Ga_xSe_z^+$ clusters identified in all parafilm coated and non-coated thin films (A-G).

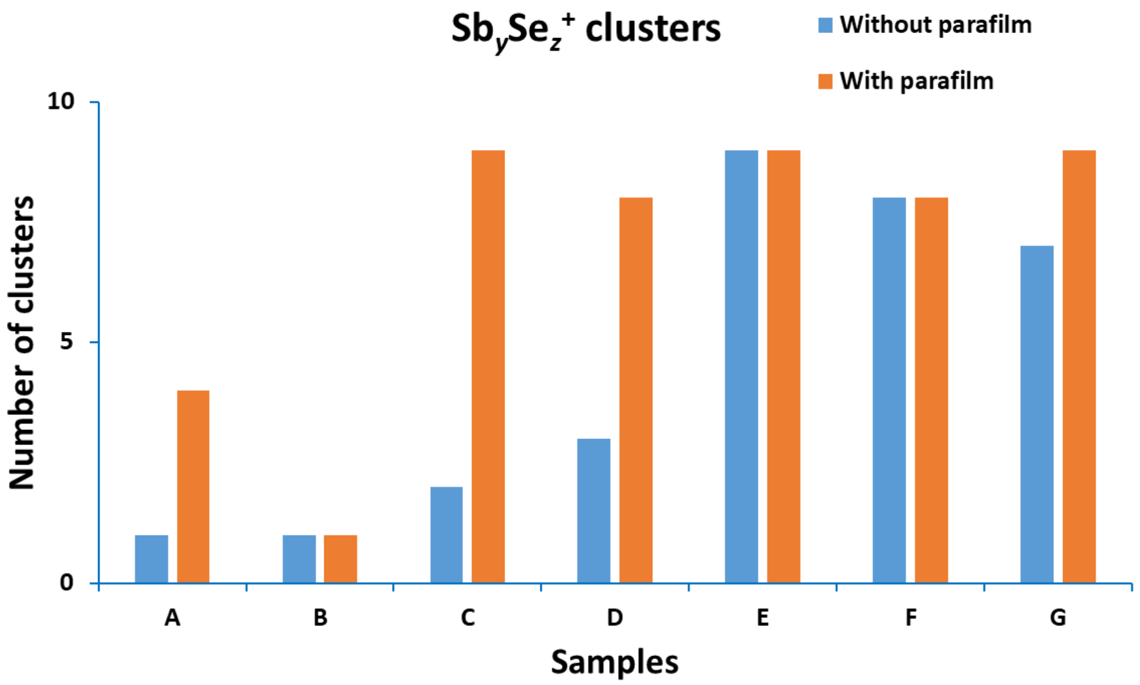

Supplementary Figure S3b: The number of binary $Sb_ySe_z^+$ clusters identified in all parafilm coated and non-coated thin films (A-G).

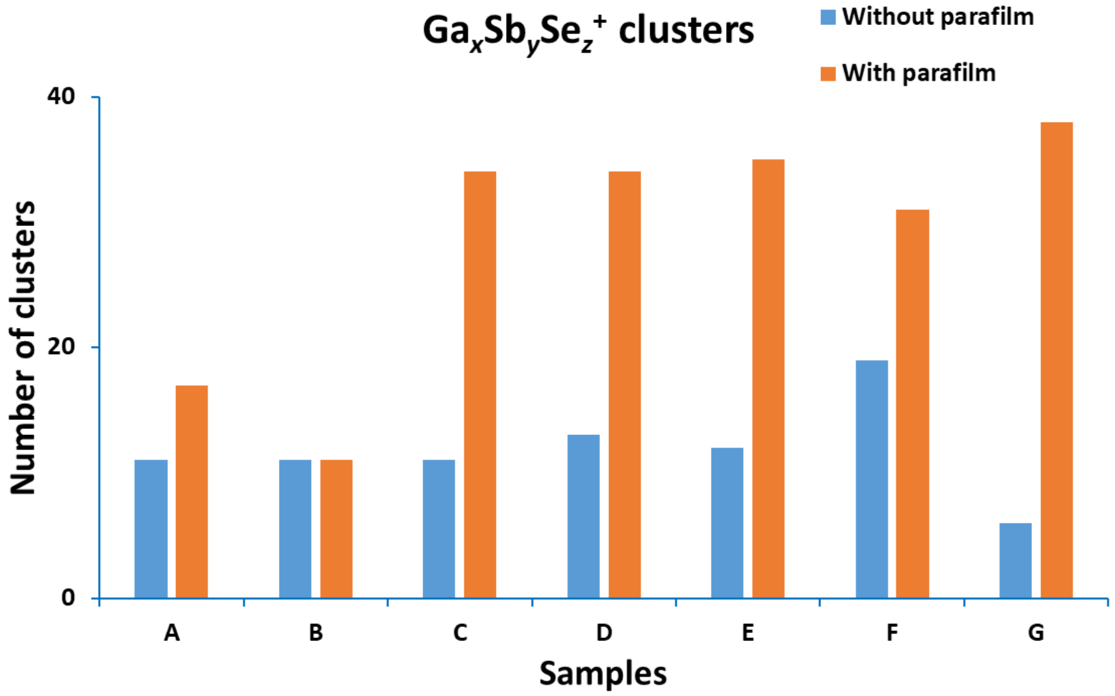

Supplementary Figure S3c: The number of binary $Sb_ySe_z^+$ clusters identified in all parafilm coated and non-coated thin films (A-G).

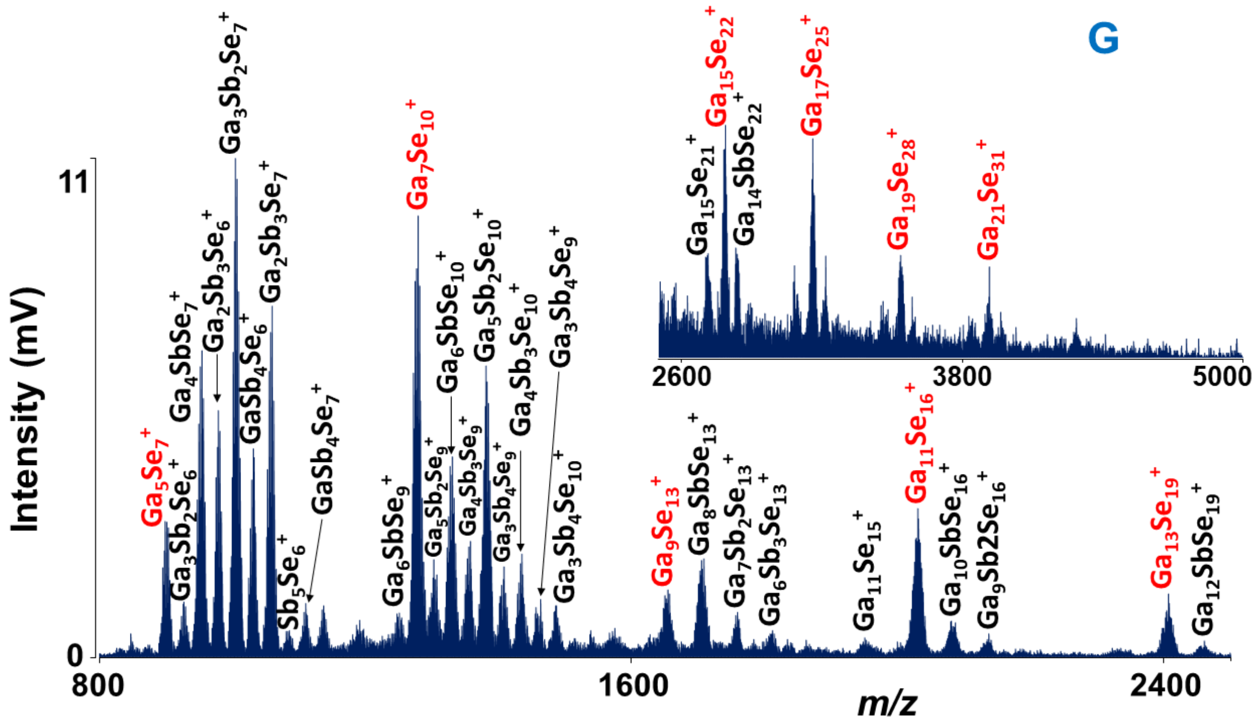

Supplementary Figure S4: Mass spectrum obtained from parafilm coated thin film of sample G. Conditions: positive ion mode, laser energy 160 a.u. A series of binary $Ga_xSe_z^+$ clusters is shown in red color.

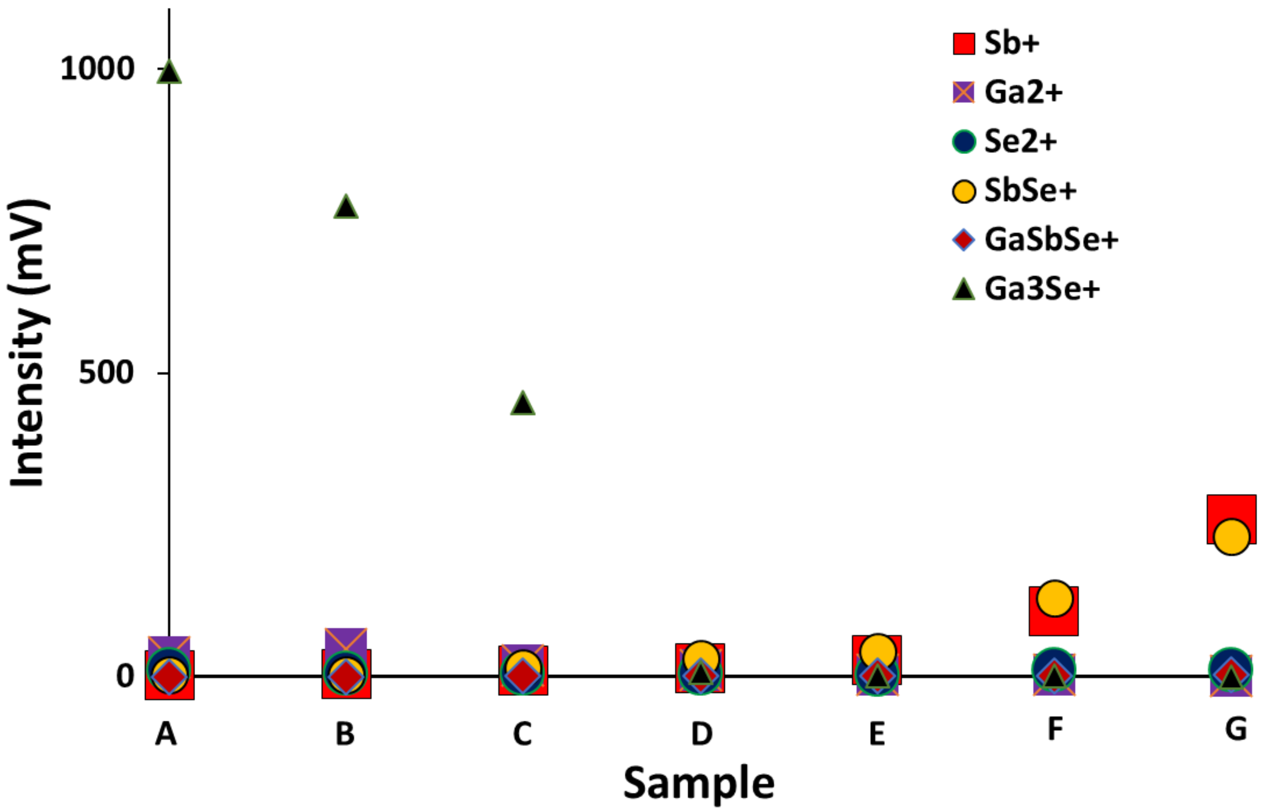

Supplementary Figure S5: The change in intensity of selected common peaks identified in all non-coated Ga-Sb-Se thin films.

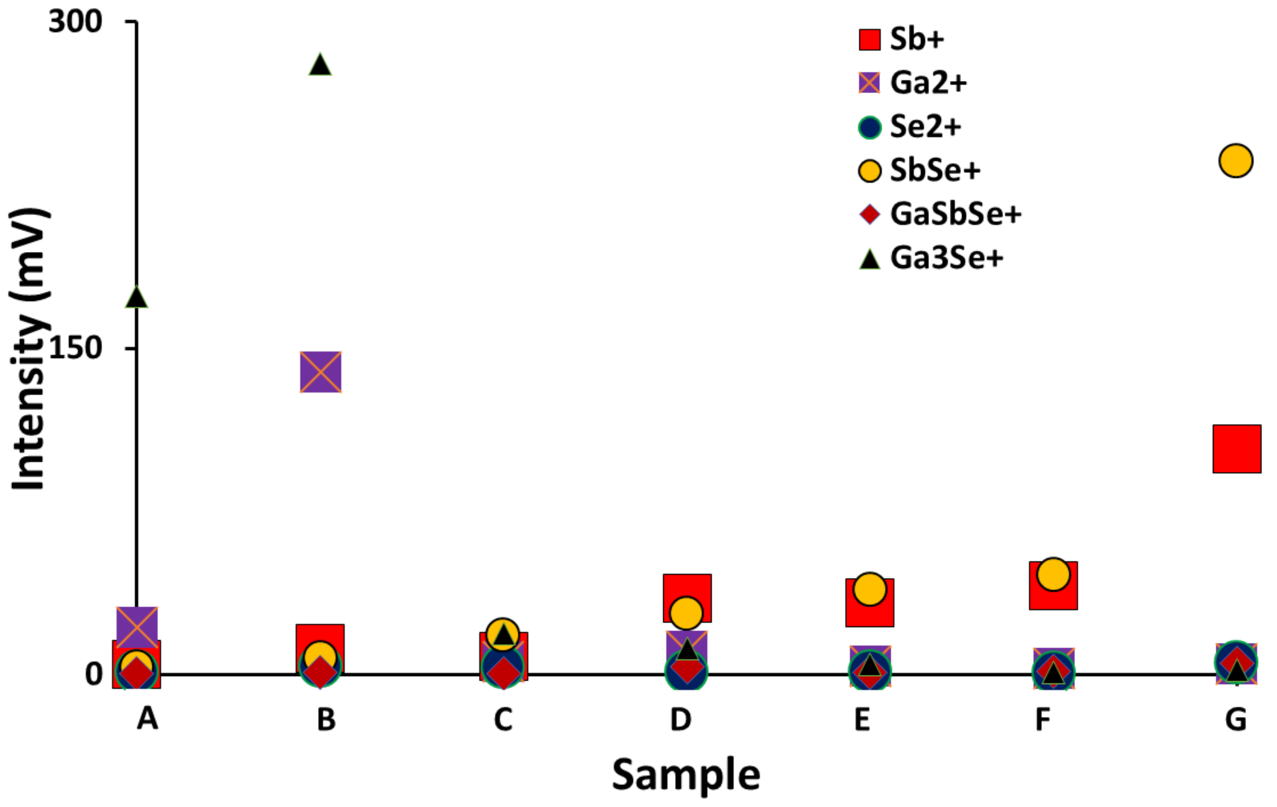

Supplementary Figure S6: The change in intensity of selected common peaks identified in all parafilm coated Ga-Sb-Se thin films.

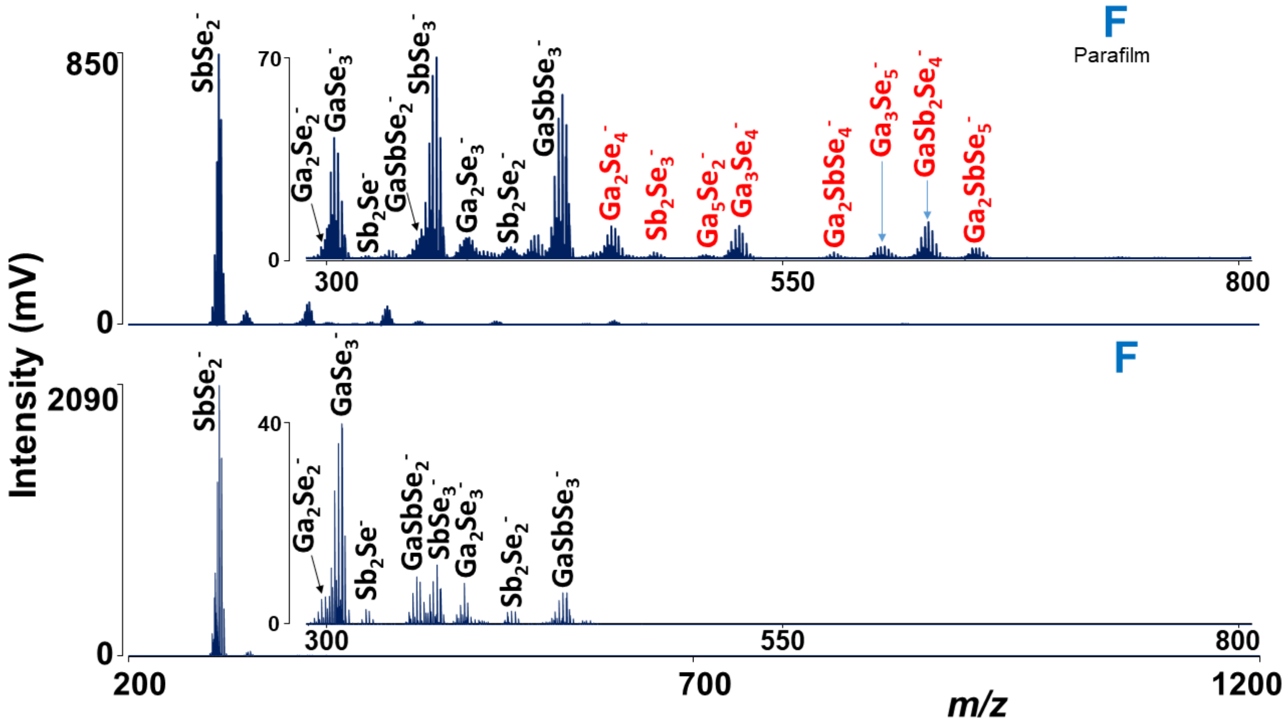

Supplementary Figure S7: Comparison of mass spectra obtained from Sample F with and without parafilm coating. The new clusters detected in mass spectra of parafilm coated thin films are shown in red. Conditions: negative ion mode, laser energy 160 a.u.

Supplementary Table S1: Overview of $Ga_xSe_z^+$, $Sb_ySe_z^+$, and $Ga_xSb_ySe_z^+$ clusters generated by LDI from Ga-Sb-Se thin films. The clusters shown in bold letters are observed in both cases (with and without using parafilm coating), clusters in italic font are detected without using parafilm coating. The clusters detected in parafilm coated thin films are shown in red.

**Sample A**	**Sample B**	**Sample C**	**Sample D**	**Sample E**	**Sample F**	**Sample G**
**Sb^+**	**Sb^+**	**Sb^+**	**Sb^+**	**Sb^+**	**Sb^+**	**Sb^+**
**Ga_2^+**	**Ga_2^+**	**Ga_2^+**	**Ga_2^+**	**Ga_2^+**	**Ga_2^+**	**Ga_2^+**
**Se_2^+**	**Se_2^+**	**Se_2^+**	**Se_2^+**	**Se_2^+**	**Se_{2+}**	**Se_2^+**
**$SbSe^+$**	**$SbSe^+$**	**$SbSe^+$**	**$SbSe^+$**	**$SbSe^+$**	**$SbSe^+$**	**$SbSe^+$**
Sb_2Se^+	**$GaSbSe^+$**	Sb_2Se^+	**Sb_2Se^+**	*$SbSe_3^+$*	**Sb_2Se^+**	**Sb_2Se^+**
$Sb_2Se_2^+$	*$GaSb_5Se_{13}^+$*	**$Sb_2Se_2^+$**	$Sb_2Se_2^+$	*$SbSe_5^+$*	**$Sb_2Se_2^+$**	**$Sb_2Se_2^+$**
Sb_3Se^+	*$Ga_2Sb_3Se_9^+$*	Sb_3Se^+	**Sb_3Se^+**	**Sb_2Se^+**	**Sb_3Se^+**	**Sb_3Se^+**
**$GaSbSe^+$**	*$Ga_2Sb_3Se_{10}^+$*	$Sb_3Se_2^+$	$Sb_3Se_2^+$	**$Sb_2Se_2^+$**	**$Sb_3Se_2^+$**	**$Sb_3Se_2^+$**
$GaSbSe_2^+$	**$Ga_3H_2^+$**	$Sb_3Se_3^+$	$Sb_3Se_3^+$	*$Sb_2Se_4^+$*	**$Sb_3Se_3^+$**	**$Sb_3Se_3^+$**
$GaSb_2Se^+$	**Ga_3Se+**	$Sb_3Se_4^+$	$Sb_3Se_4^+$	**Sb_3Se^+**	**$Sb_3Se_4^+$**	$Sb_3Se_4^+$
$GaSb_2Se_2^+$	**$Ga_3Se_2^+$**	$Sb_5Se_4^+$	$Sb_5Se_6^+$	$Sb_3Se_2^+$	**$Sb_5Se_4^+$**	$Sb_5Se_4^+$
$GaSb_2Se_3^+$	$Ga_3Sb_2Se_7^+$	$Sb_5Se_6^+$	**$GaSbSe^+$**	**$Sb_3Se_3^+$**	**$GaSbSe^+$**	$Sb_5Se_6^+$
$GaSb_2Se_4^+$	**$Ga_4SbSe_7^+$**	**$GaSbSe^+$**	**$GaSbSe_2^+$**	**$Sb_3Se_4^+$**	**$GaSbSe_2^+$**	**$GaSbSe^+$**
$GaSb_4Se_5^+$	*$Ga_4Sb_3Se_8^+$*	$GaSbSe_2^+$	$GaSb_2Se^+$	$Sb_5Se_4^+$	**$GaSb_2Se^+$**	$GaSbSe_2^+$
$Ga_2Se_2^+$	**$Ga_5Se_4^+$**	$GaSb_2Se^+$	$GaSb_2Se_2^+$	$Sb_5Se_6^+$	**$GaSb_2Se_2^+$**	**$GaSb_2Se^+$**
$Ga_2SbSe_2^+$	**$Ga_5Se_7^+$**	$GaSb_2Se_2^+$	$GaSb_2Se_3^+$	**$GaSbSe^+$**	**$GaSb_2Se_3^+$**	**$GaSb_2Se_2^+$**
$Ga_2SbSe_4^+$	$Ga_5Sb_2Se_9^+$	$GaSb_2Se_3^+$	**$GaSb_2Se_4^+$**	**$GaSbSe_2^+$**	**$GaSb_2Se_4^+$**	$GaSb_2Se_3^+$
$Ga_2Sb_3Se_3^+$	$Ga_5Sb_2Se_{10}^+$	$GaSb_2Se_4^+$	$GaSb_2Se_5^+$	**$GaSb_2Se^+$**	**$GaSb_2Se_5^+$**	**$GaSb_2Se_4^+$**
$Ga_2Sb_3Se_6^+$	*Ga_6Se^+*	$GaSb_2Se_5^+$	$GaSb_4Se_5^+$	$GaSb_2Se_2^+$	**$GaSb_4Se_5^+$**	$GaSb_2Se_5^+$
**$Ga_3H_2^+$**	**$Ga_6SbSe_9^+$**	$GaSb_4Se_5^+$	$GaSb_4Se_6^+$	**$GaSb_2Se_3^+$**	**$GaSb_4Se_6^+$**	$GaSb_4Se_5^+$
**Ga_3Se^+**	**$Ga_6SbSe_{10}^+$**	$GaSb_4Se_6^+$	$Ga_2SbSe_2^+$	$GaSb_2Se_4^+$	$GaSb_4Se_7^+$	$GaSb_4Se_6^+$
**$Ga_3Se_2^+$**	**$Ga_7Se_3^+$**	**$Ga_2SbSe_2^+$**	$Ga_2SbSe_4^+$	**$GaSb_2Se_5^+$**	**$Ga_2SbSe_2^+$**	$GaSb_4Se_7^+$
$Ga_3SbSe_2^+$	$Ga_7Se_8^+$	$Ga_2SbSe_4^+$	$Ga_2Sb_3Se_5^+$	$GaSb_4Se_5^+$	**$Ga_2SbSe_4^+$**	**$Ga_2SbSe_2^+$**
$Ga_3Sb_2Se_4^+$	**$Ga_7Se_9^+$**	$Ga_2Sb_3Se_5^+$	$Ga_2Sb_3Se_6^+$	$GaSb_4Se_6^+$	$Ga_2Sb_3Se_5^+$	$Ga_2SbSe_4^+$
$Ga_3Sb_2Se_6^+$	**$Ga_7Se_{10}^+$**	$Ga_2Sb_3Se_6^+$	$Ga_2Sb_3Se_7^+$	*$GaSe_7^+$*	**$Ga_2Sb_3Se_6^+$**	$Ga_2Sb_3Se_6^+$
$Ga_3Sb_2Se_7^+$	**$Ga_8Se_2^+$**	$Ga_2Sb_3Se_7^+$	**$Ga_3H_2^+$**	$Ga_2SbSe_2^+$	**$Ga_2Sb_3Se_7^+$**	$Ga_2Sb_3Se_7^+$
**$Ga_4SbSe_7^+$**	*$Ga_8SbSe_{11}^+$*	**$Ga_3H_2^+$**	**Ga_3Se_+**	**$Ga_2SbSe_4^+$**	$Ga_2Sb_5Se_{10}^+$	$Ga_2Sb_5Se_{10}^+$
$Ga_4SbSe_5^+$	**$Ga_8SbSe_{12}^+$**	**Ga_3Se^+**	**$Ga_3Se_2^+$**	$Ga_2Sb_3Se_5^+$	**$Ga_3H_2^+$**	**$Ga_3H_2^+$**
$Ga_4Sb_3Se_9^+$	$Ga_8SbSe_{13}^+$	**$Ga_3Se_2^+$**	$Ga_3SbSe_2^+$	$Ga_2Sb_3Se_6^+$	**Ga_3Se^+**	**Ga_3Se^+**
$Ga_5Se_4^+$	**$Ga_9Se_{12}^+$**	$Ga_3SbSe_2^+$	$Ga_3SbSe_{12}^+$	$Ga_2Sb_3Se_7^+$	**$Ga_3Se_2^+$**	**$Ga_3Se_2^+$**
**$Ga_5Se_7^+$**	**$Ga_9Se_{13}^+$**	$Ga_3Sb_2Se_4^+$	$Ga_3Sb_2Se_4^+$	**$Ga_3H_2^+$**	**$Ga_3SbSe_2^+$**	**$Ga_3SbSe_2^+$**
**$Ga_6SbSe_{10}^+$**	*$Ga_9Se_{17}^+$*	$Ga_3Sb_2Se_6^+$	$Ga_3Sb_2Se_6^+$	**Ga_3Se^+**	$Ga_3Sb_2Se_6^+$	$Ga_3Sb_2Se_7^+$
$Ga_6Sb_3Se_{13}^+$	*$Ga_{10}Se_{16}^+$*	$Ga_3Sb_2Se_7^+$	$Ga_3Sb_2Se_7^+$	**$Ga_3Se_2^+$**	**$Ga_3Sb_2Se_7^+$**	$Ga_3Sb_2Se_6^+$
**$Ga_7Se_{10}^+$**	$Ga_{10}SbSe_{16}^+$	$Ga_3Sb_4Se_9^+$	$Ga_3Sb_4Se_9^+$	**$Ga_3SbSe_2^+$**	$Ga_3Sb_4Se_9^+$	$Ga_3Sb_4Se_9^+$

$Ga_{8}SbSe_{11}^{+}$	**$Ga_{11}Se_{14}^{+}$**	$Ga_{4}SbSe_{5}^{+}$	$Ga_{4}SbSe_{7}^{+}$	$Ga_{3}Sb_{2}Se_{4}^{+}$	$Ga_{3}Sb_{4}Se_{10}^{+}$	$Ga_{3}Sb_{4}Se_{10}^{+}$
$Ga_{8}SbSe_{12}^{+}$	**$Ga_{11}Se_{15}^{+}$**	$Ga_{4}SbSe_{6}^{+}$	$Ga_{4}Sb_{3}Se_{9}^{+}$	$Ga_{3}Sb_{2}Se_{6}^{+}$	$Ga_{4}SbSe_{6}^{+}$	**$Ga_{4}SbSe_{7}^{+}$**
**$Ga_{8}SbSe_{13}^{+}$**	**$Ga_{11}Se_{16}^{+}$**	**$Ga_{4}SbSe_{7}^{+}$**	$Ga_{4}Sb_{3}Se_{10}^{+}$	$Ga_{3}Sb_{2}Se_{7}^{+}$	**$Ga_{4}SbSe_{7}^{+}$**	$Ga_{4}Sb_{3}Se_{9}^{+}$
$Ga_{9}Se_{11}^{+}$	$Ga_{12}SbSe_{19}^{+}$	$Ga_{4}Sb_{3}Se_{9}^{+}$	*$Ga_{4}Sb_{3}Se_{12}^{+}$*	$Ga_{3}Sb_{4}Se_{9}^{+}$	$Ga_{4}Sb_{3}Se_{9}^{+}$	$Ga_{4}Sb_{3}Se_{10}^{+}$
**$Ga_{9}Se_{12}^{+}$**	$Ga_{13}Se_{17}^{+}$	$Ga_{4}Sb_{3}Se_{10}^{+}$	**$Ga_{5}Se_{7}^{+}$**	$Ga_{4}SbSe_{5}^{+}$	$Ga_{4}Sb_{3}Se_{10}^{+}$	**$Ga_{5}Se_{7}^{+}$**
**$Ga_{9}Se_{13}^{+}$**	$Ga_{13}Se_{18}^{+}$	*$Ga_{4}Sb_{3}Se_{12}^{+}$*	$Ga_{5}Sb_{2}Se_{9}^{+}$	**$Ga_{4}SbSe_{7}^{+}$**	$Ga_{5}Se_{4}^{+}$	$Ga_{5}Sb_{2}Se_{9}^{+}$
$Ga_{10}SbSe_{16}^{+}$	**$Ga_{13}Se_{19}^{+}$**	$Ga_{5}Se_{4}^{+}$	**$Ga_{5}Sb_{2}Se_{10}^{+}$**	$Ga_{4}Sb_{3}Se_{9}^{+}$	**$Ga_{5}Se_{7}^{+}$**	$Ga_{5}Sb_{2}Se_{10}^{+}$
**$Ga_{11}Se_{14}^{+}$**	*$Ga_{14}SbSe_{9}^{+}$*	**$Ga_{5}Se_{7}^{+}$**	**$Ga_{6}SbSe_{9}^{+}$**	$Ga_{4}Sb_{3}Se_{10}^{+}$	$Ga_{5}Sb_{2}Se_{9}^{+}$	$Ga_{6}SbSe_{9}^{+}$
**$Ga_{11}Se_{15}^{+}$**	$Ga_{15}Se_{21}^{+}$	*$Ga_{5}Se_{11}^{+}$*	**$Ga_{6}SbSe_{10}^{+}$**	*$Ga_{4}Sb_{3}Se_{12}^{+}$*	**$Ga_{5}Sb_{2}Se_{10}^{+}$**	$Ga_{6}SbSe_{10}^{+}$
**$Ga_{11}Se_{16}^{+}$**	**$Ga_{15}Se_{22}^{+}$**	$Ga_{5}Sb_{2}Se_{9}^{+}$	*$Ga_{6}Sb_{3}Se_{13}^{+}$*	**$Ga_{5}Se_{7}^{+}$**	**$Ga_{6}SbSe_{9}^{+}$**	$Ga_{6}Sb_{3}Se_{13}^{+}$
$Ga_{13}Se_{18}^{+}$	$Ga_{17}Se_{24}^{+}$	**$Ga_{5}Sb_{2}Se_{10}^{+}$**	**$Ga_{7}Se_{10}^{+}$**	$Ga_{5}Sb_{2}Se_{9}^{+}$	**$Ga_{6}SbSe_{10}^{+}$**	$Ga_{7}Se_{10}^{+}$
**$Ga_{13}Se_{19}^{+}$**	**$Ga_{17}Se_{25}^{+}$**	**$Ga_{6}SbSe_{9}^{+}$**	$Ga_{7}Sb_{2}Se_{13}^{+}$	$Ga_{5}Sb_{2}Se_{10}^{+}$	**$Ga_{7}Se_{10}^{+}$**	$Ga_{7}Sb_{2}Se_{13}^{+}$
$Ga_{15}Se_{22}^{+}$	$Ga_{19}Se_{28}^{+}$	**$Ga_{6}SbSe_{10}^{+}$**	*$Ga_{8}SbSe_{11}^{+}$*	$Ga_{6}SbSe_{9}^{+}$	**$Ga_{7}Sb_{2}Se_{13}^{+}$**	$Ga_{8}SbSe_{13}^{+}$
$Ga_{17}Se_{25}^{+}$		*$Ga_{6}Sb_{3}Se_{13}^{+}$*	**$Ga_{8}SbSe_{12}^{+}$**	**$Ga_{6}SbSe_{10}^{+}$**	**$Ga_{8}SbSe_{13}^{+}$**	$Ga_{9}Se_{13}^{+}$
$Ga_{19}Se_{28}^{+}$		*$Ga_{7}Se_{9}^{+}$*	**$Ga_{8}SbSe_{13}^{+}$**	$Ga_{6}Sb_{3}Se_{13}^{+}$	**$Ga_{9}Se_{13}^{+}$**	$Ga_{9}Sb_{2}Se_{16}^{+}$
		**$Ga_{7}Se_{10}^{+}$**	**$Ga_{9}Se_{12}^{+}$**	**$Ga_{7}Se_{10}^{+}$**	$Ga_{11}Se_{16}^{+}$	$Ga_{10}SbSe_{16}^{+}$
		$Ga_{7}Se_{11}^{+}$	**$Ga_{9}Se_{13}^{+}$**	$Ga_{7}Sb_{2}Se_{13}^{+}$	$Ga_{13}Se_{19}^{+}$	$Ga_{11}Se_{15}^{+}$
		$Ga_{7}Sb_{2}Se_{13}^{+}$	**$Ga_{10}SbSe_{16}^{+}$**	$Ga_{8}SbSe_{12}^{+}$	$Ga_{15}Se_{22}^{+}$	$Ga_{11}Se_{16}^{+}$
		$Ga_{8}SbSe_{11}^{+}$	*$Ga_{11}Se_{14}^{+}$*	**$Ga_{8}SbSe_{13}^{+}$**	$Ga_{17}Se_{25}^{+}$	$Ga_{12}SbSe_{19}^{+}$
		$Ga_{8}SbSe_{12}^{+}$	**$Ga_{11}Se_{15}^{+}$**	$Ga_{9}Se_{12}^{+}$	$Ga_{19}Se_{28}^{+}$	$Ga_{13}Se_{19}^{+}$
		**$Ga_{8}SbSe_{13}^{+}$**	**$Ga_{11}Se_{16}^{+}$**	**$Ga_{9}Se_{13}^{+}$**		$Ga_{14}SbSe_{22}^{+}$
		$Ga_{9}Se_{11}^{+}$	$Ga_{12}SbSe_{19}^{+}$	$Ga_{9}Sb_{2}Se_{16}^{+}$		$Ga_{15}Se_{21}^{+}$
		**$Ga_{9}Se_{12}^{+}$**	**$Ga_{13}Se_{18}^{+}$**	**$Ga_{10}SbSe_{16}^{+}$**		$Ga_{15}Se_{22}^{+}$
		**$Ga_{9}Se_{13}^{+}$**	**$Ga_{13}Se_{19}^{+}$**	$Ga_{11}Se_{15}^{+}$		$Ga_{17}Se_{25}^{+}$
		**$Ga_{10}SbSe_{16}^{+}$**	$Ga_{14}SbSe_{22}^{+}$	**$Ga_{11}Se_{16}^{+}$**		$Ga_{19}Se_{28}^{+}$
		$Ga_{11}Se_{14}^{+}$	$Ga_{15}Se_{21}^{+}$	$Ga_{12}SbSe_{19}^{+}$		
		**$Ga_{11}Se_{15}^{+}$**	$Ga_{15}Se_{22}^{+}$	$Ga_{13}Se_{19}^{+}$		
		**$Ga_{11}Se_{16}^{+}$**	$Ga_{17}Se_{25}^{+}$	$Ga_{15}Se_{21}^{+}$		
		$Ga_{12}SbSe_{19}^{+}$	$Ga_{19}Se_{28}^{+}$	$Ga_{15}Se_{22}^{+}$		
		**$Ga_{13}Se_{18}^{+}$**		$Ga_{17}Se_{25}^{+}$		
		**$Ga_{13}Se_{19}^{+}$**		$Ga_{19}Se_{28}^{+}$		
		$Ga_{15}Se_{22}^{+}$				
		$Ga_{17}Se_{25}^{+}$				

Supplementary Table S2: Overview of $Ga_xSe_z^-$, $Sb_ySe_z^-$, and $Ga_xSb_ySe_z^-$ clusters generated by LDI from Ga-Sb-Se thin films. The clusters shown in bold letters are observed in both cases (with and without using parafilm coating), clusters in italic font are detected without using parafilm coating. The clusters detected in parafilm coated thin films are shown in red.

**Sample A**	**Sample B**	**Sample C**	**Sample D**	**Sample E**	**Sample F**	**Sample G**
**Se_2^-**	**Se_2^-**	**Se_2^-**	**Se_2^-**	**Se_2^-**	**Se_2^-**	**Se_2^-**
Se_4^-	Se_4^-	Se_4^-	**$SbSe^+$**	**$SbSe^-$**	**$SbSe^-$**	Se_4^-
Se_5^-	**$SbSe^-$**	$SbSe^-$	**$SbSe_2^+$**	**$SbSe_2^-$**	**$SbSe_2^-$**	**$SbSe^-$**
**$SbSe^-$**	**$SbSe_2^-$**	**$SbSe_2^-$**	**$SbSe_3^+$**	**$SbSe_3^-$**	**$SbSe_3^-$**	**$SbSe_2^-$**
**$SbSe_2^-$**	**$SbSe_3^-$**	**$SbSe_3^-$**	**$Sb_2Se_2^+$**	**$Sb_2Se_2^-$**	**Sb_2Se^-**	**$SbSe_3^-$**
**$SbSe_3^-$**	*$Sb_3Se_7^-$*	**$Sb_2Se_2^-$**	**$GaSe^+$**	**$GaSe^-$**	$Sb_2Se_2^-$	*Sb_2Se^-*
**$GaSe^-$**	*$Sb_3Se_{11}^-$*	**$GaSe^-$**	**$GaSe_2^-$**	**$GaSe_2^-$**	$Sb_2Se_3^-$	$Sb_2Se_2^-$
**$GaSe_2^-$**	**$GaSe^-$**	**$GaSe_2^-$**	**$GaSe_3^-$**	**$GaSe_3^-$**	*$GaSe^-$*	$Sb_2Se_3^-$
**$GaSe_3^-$**	**$GaSe_2^-$**	**$GaSe_3^-$**	**$GaSe_4^-$**	**$GaSe_4^-$**	*$GaSe_2^-$*	$Sb_3Se_4^-$
**$GaSbSe_2^-$**	**$GaSe_3^-$**	**$GaSbSe_2^-$**	**$GaSbSe_2^-$**	**$GaSbSe_2^-$**	**$GaSe_3^-$**	*$GaSe^-$*
**$GaSbSe_3^-$**	*$GaSbSe^-$*	**$GaSbSe_3^-$**	**$GaSbSe_3^-$**	**$GaSbSe_3^-$**	**$GaSbSe_2^-$**	*$GaSe_2^-$*
**$Ga_2Se_2^-$**	*$GaSbSe_2^-$*	$GaSb_2Se_4^-$	**$Ga_2Se_2^-$**	$GaSb_2Se_4^-$	**$GaSbSe_3^-$**	**$GaSe_3^-$**
**$Ga_2Se_3^-$**	*$GaSbSe_3^-$*	**$Ga_2Se_2^-$**	**$Ga_2Se_3^-$**	**$Ga_2Se_2^-$**	$GaSb_2Se_4^-$	**$GaSbSe_2^-$**
$Ga_2Se_4^-$	*$GaSbSe_4^-$*	**$Ga_2Se_3^-$**	*$Ga_2Se_4^-$*	**$Ga_2Se_3^-$**	$GaSb_2Se_2^-$	$GaSbSe_3^-$
$Ga_2Sb_3Se_4^-$	*$Ga_2Se_2^-$*	*$Ga_3Se_3^-$*	$Ga_2SbSe_4^-$	$Ga_2SbSe_4^-$	$GaSb_2Se_3^-$	$GaSb_2Se_4^-$
**$Ga_3Se_3^-$**	*$Ga_2Se_3^-$*	**$Ga_3Se_4^-$**	*$Ga_3Se_3^-$*	$Ga_2SbSe_5^-$	**$Ga_2Se_2^-$**	$GaSb_2Se_5^-$
$Ga_3Se_4^-$	*$Ga_2Sb_3Se_6^-$*	$Ga_5Se_2^-$	*$Ga_3Se_4^-$*	$Ga_3Se_4^-$	**$Ga_2Se_3^-$**	$GaSb_4Se_6^-$
$Ga_3Se_5^-$	*$Ga_3Se_3^-$*		$Ga_3Se_5^-$	$Ga_3Se_5^-$	$Ga_2Se_4^-$	**$Ga_2Se_2^-$**
$Ga_3Sb_2Se_4^-$	$Ga_3Se_4^-$			$Ga_3Sb_2Se_8^-$	$Ga_2SbSe_4^-$	**$Ga_2Se_3^-$**
$Ga_5Se_7^-$	*$Ga_3Se_5^-$*			$Ga_4SbSe_7^-$	$Ga_2SbSe_5^-$	$Ga_2Se_4^-$
	$Ga_3Sb_4Se_9^-$			$Ga_4SbSe_8^-$	$Ga_3Se_4^-$	$Ga_2SbSe_4^-$
	$Ga_4Se_6^-$			$Ga_4SbSe_9^-$	$Ga_3Se_5^-$	$Ga_2SbSe_5^-$
	$Ga_4Se_7^-$			$Ga_5Se_7^-$	$Ga_5Se_2^-$	$Ga_3Se_4^-$
	$Ga_4SbSe_8^-$			$Ga_5Se_9^-$		$Ga_3Se_5^-$
	$Ga_4SbSe_{11}^-$			$Ga_7Se_5^-$		$Ga_3Sb_2Se_8^-$
	$Ga_4Sb_3Se_9^-$					$Ga_5Se_2^-$
	**$Ga_5Se_6^-$**					$Ga_7Se_5^-$
	**$Ga_5Se_7^-$**					
	$Ga_5Se_8^-$					
	**$Ga_5Se_9^-$**					
	$Ga_5Se_{12}^-$					
	$Ga_6Sb_3Se_{13}^-$					

	$Ga_8SbSe_{14}^-$					
	$Ga_9Se_{14}^-$					
	$Ga_{13}Se_{20}^-$					
	$Ga_{15}Se_{23}^-$					
	$Ga_{17}Se_{26}^-$					
